# How much do antiretroviral drugs penetrate into the central nervous system?

**Published:** 2011-11-24

**Authors:** L Ene, D Duiculescu, SM Ruta

**Affiliations:** * “Dr. Victor Babes” Hospital for Infectious and Tropical Diseases, 281 Mihai Bravu Ave., District 3, 030303, Bucharest, Romania; ** “Carol Davila” University of Medicine and Pharmacy and “St. S. Nicolau” Institute of Virology, 285 Mihai Bravu Ave., District 3, 030304, Bucharest, Romania

**Keywords:** antiretroviral treatment, central nervous system, penetrability, HIV

## Abstract

The central nervous system can act as a compartment in which HIV can replicate independently from plasma, and also as a sanctuary in which, under suboptimal drug pressure, HIV antiretroviral genetic variants can occur. Continuous replication of HIV in brain can contribute to neurocognitive impairment. Therefore, reaching adequate concentrations of antiretrovirals in the central nervous system might be essential in providing neuroprotection and improving neurocognition. Antiretrovirals have a restricted entry into the brain, due to several factors: the unique structure of the blood-brain barrier, and the existence of efficient efflux mechanisms. However, there is a high variability of antiretrovirals in reaching therapeutic drug concentrations in cerebrospinal fluid, that depend on the characteristics of the antiretrovirals (molecular weight, lipophilicity, protein binding) and on their capacity to be substrate for efflux transporters. The review aims to discuss the main mechanisms that interfere with antiretroviral penetration into central nervous system, and to summarize the current data concerning the penetrability of different antiretrovirals into the cerebrospinal fluid.

**Abbreviations:** ART = antiretroviral treatment; ARV = antiretrovirals; NRTI = nucleos(t)idic reverse-transcriptase inhibitors; NNRTI = non-nucleosidic reverse transcriptase inhibitors; INNRT = integrase inhibitors; CNS = central nervous system; BBB = blood-brain barrier; CMT = carrier-mediated transport; AET = active efflux transports; PGP = P-glycoprotein; MRP = multidrug resistance-associated proteins; SLC = solute carriers; OATP = organic anion transporting polypeptide; OAT = organic anion transporters; OCT = organic cation transporters; EFV = Efavirenz; IDV = Indinavir; ZDV = Zidovudine; d4T = Stavudine; ABC = Abacavir; ddI = Didanosine; 3TC = Lamivudine; TDF = Tenofovir; NVP = Nevirapine; PI = Protease inhibitors; APV = Amprenavir; NFV = Nelfinavir; SQV = Saquinavir; ATV = Ataznavir; TPV = Tipranavir; DRV = Darunavir; T20 = Enfuvirtide; RGV = Raltegravir

## Introduction

Antiretroviral treatment (ART) represents a keystone in the evolution of HIV–infection by reducing mortality, increasing life span and quality. Nowadays, antiretrovirals (ARV) from six classes are available: nucleos(t)idic reverse–transcriptase inhibitors (NRTI), that interfere with reverse transcriptase by competing with the natural substrates and incorporating into viral DNA to act as chain terminators in the synthesis of proviral DNA; non–nucleosidic reverse transcriptase inhibitors (NNRTI)– that bind directly to the catalytic site of the reverse transcriptase, protease inhibitors (PI)– that inhibit the proteolitic cleavage of polipeptridic precursors, giving rise to noninfectious viral particles; integrase inhibitors (INNRT) that block the integration of proviral DNA in the cell DNA, CCR5 co–receptor antagonists that prevent interaction of the V3 loop of gp120 with the CCR5 coreceptor and fusion inhibitors that block entry of HIV into the target cell. Effectiveness of ART regimens is usually evaluated by measuring HIV RNA levels in plasma. However, there is evidence that HIV can replicate in compartments distinct from plasma [**1**] and can establish viral sanctuaries, with limited penetrations of antiretrovirals where viral replication continues during treatment, and ultimately determines occurrence of resistant HIV viral strains [**2**].

The central nervous system (CNS) is one of the compartments in which HIV determines an autonomous infection since the early stages of infection, but it is also a sanctuary in which HIV can independently replicate and has a genetic profile distinct from plasma, due to an inadequate concentration of ARV [**3,4**]. Neurocognitive impairment can be the result of HIV replication in the CNS, even in patients with suppressed plasma viral loads [**5**] and can interfere with the patient’s functionality [**6**].

There seems to be a direct correlation between the concentrations of ARV in the CSF and the decrease of HIV CSF viral load [**7,8**]. Letendre developed and improved a quantification rank of antiretrovirals in CSF, which can be a useful tool for physicians in selecting the adequate ART for patients with neurocognitive impairment [**8,9**].

This article intends to briefly present the factors that contribute to different concentrations of ARV in the CSF, and the current data concerning the penetrability of antiretrovirals in the CSF.

### Factors that influence the penetration of drugs across the blood-brain barrier

The access of various molecules into the CNS is closely related to their ability to penetrate through the blood-brain barrier (BBB). The BBB consists of several compounds, two of which are better described: the vascular BBB primarily includes the cerebral capillaries and the endothelial cells sealed by tight junctions and the blood-CSF barrier represented mainly by the choroid plexuses, which form the interface between the blood and the ventricular cerebrospinal fluid [**10**].

The penetrability of different drugs including ARV across the BBB is related to several characteristics like their physical and chemical particularities, their molecular weight, the protein binding, their lipid solubility, the degree of ionization, the molecular pumps mechanisms and also to the cerebral blood flow and the degree of local inflammation.

Diffusion is one of the most common mechanisms of crossing the BBB [**11**]. Low molecular weight and higher lipid solubility facilitate a better penetration through the BBB. Conversely, high protein binding rates associated with low free drug levels diminish the penetration through the BBB.

The efflux or influx transport mechanisms also play a crucial role in drug penetration into the CNS. The transporters are classified as carrier–mediated transport (CMT), active efflux transports (AET), and receptor–mediated transport (RMT) [**12**]. CMT systems are generally responsible for the transport of nutrients (glucose, amino–acids) across the BBB. RMT are used to carry endogenous large–molecule neuro–peptides, mainly hormones, into the brain by specialized ligand–specific receptor systems, including the insulin receptor, the transferrin receptor, the insulin–like growth factor receptor, the leptin receptor, the neonatal Fc receptor or the type BI scavenger receptor [**13**]. However, the most important transporter mechanisms from the ARV perspective are the AET. Active efflux transporters limit the brain uptake of several high lipophilic drugs [**14**]. P–glycoprotein (PGP), multidrug resistance–associated proteins (MRP) are the best described energy–dependent efflux transporters that represent a major obstacle for ARV penetration in CNS. Solute carriers (SLC) are another group of efflux transporters especially designated for anions [**15**]. Members of this group include organic anion transporting polypeptide (OATP), organic anion transporters (OAT) and organic cation transporters (OCT). All these polypeptides are Na– and ATP–independent and they are expressed on the cell membrane of the endothelial capillaries in the brain. Choroid plexuses are also significantly involved in the cellular uptake of drugs, including ARV in the brain [**16**].

### Penetrability of ARV in CNS

Antiretrovirals are following the same rules regarding their penetration in CNS. **Table 1** shows the molecular weight, percentage of protein binding, range of plasma and CSF concentration.

**Table 1:** Main properties of antiretrovirals

Antiretroviral	Molecular weight	Protein binding %	Plasma concentration	CSF concentration
Nucleos(t)ide revers–transcriptase inhibitors
Zidovudine (ZDV)	267.2	34–38	4.5–6.7 μmol/ml	0.12–0.41 μmol/ml
Lamivudine (3TC)	229.3	<36	4.3–8.7 μmol/ml	0.05––1.14 μmol/ml
Stavudine (D4T)	224.2	Negligible	3.3–6.4 μmol/ml	0.2–0.36 μmol/ml
Didanosine (DDI)	236.2	<5	2.12–11 μmol/ml	0.17–0.51 μmol/ml
Abacavir (ABC)	286.3	49	5.2–10.9 μmol/ml	0.5–1.8 μmol/ml
Tenofovir disoproxil –TDF (PMPA precursor of TDF)	519.4
	289.2	–(PMPA)
	(PMPA)
**Non–nucleosidic revers–transcriptase inhibitors**
Nevirapine (NVP)	266.3	60	7.5–16.9 μmol/ml	1.3–10.9 μmol/ml
Efavirenz (EFV)	315.7	99.5	9.2–16.6 μmol/ml	0.006–0.09 μmol/ml
Etravirine (ETV)	435	99.9	0,6 μmol/ml	
**Protease inhibitors**
Indinavir (IDV)	613.8	60	12.2–13.0 μmol/ml	0.03–0.66 μmol/ml
Ritonavir (RTV)	721	98–99	10.5–26 μmol/ml	Nd–0.32 μmol/ml
Nelfinavir (NFV)	567.8	>99	5.6–8.45 μmol/ml	Nd–0.012 μmol/ml
Saquinavir (SQV)	670.9	98	1.84–3.23 μmol/ml	Nd–0.008 μmol/ml
Amprenavir (APV)	505.6	90	10.6–19.2 μmol/ml	Nd–0.36 μmol/ml
Lopinavir (LPV)	628.8	98–99	67945 ± 4215 μg/l	16.75 ± 8.6 μg/l
Atazanavir (ATV)	704.9	++(+)	128–6200 ng/ml	Nd–40 ng/ml
Fosamprenavir – FPV(conveted rapidely to APV)	585.6	+++		
Darunavir (DRV)	548	95	1800–12900 ng/ml	15.9–212.0 ng/ml
**Entry inhibitors**
Enfuvirtide (T20)	446.2	+++	3.69 (SD 1.83) μg/mL	Nd
**Inhibitors of CCR5 co–receptors**
Maraviroc (MVC)	514	76	21.4–478.0 ng/ml	1.83–12.2 ng/ml
**Integrase inhibitors**
Raltegravir (RGV)	444	83	37–5180 ng/ml	2.0–126 ng/ml

The levels of ART in the CSF are low compared to plasma. Nevertheless, the question is if these levels are enough to inhibit the replication of HIV in the CSF. Most studies use the half maximal inhibitory concentration (IC50) of the ART for the wild type HIV as reference. IC50, used for in vitro is comparable to the half maximal effective concentration (EC50) that represents the plasma concentration required for obtaining 50% of a maximum in vivo effect. However, IC90 and IC95 seem to be a better reference for the effectiveness of a specific drug.

**Table 1** shows that NRTI as class have the advantage of a good CSF concentration: low molecular weight and the lowest rates of protein binding. A study of NRTI penetrability in the CNS, based on sample collection at different time-points demonstrated that zidovudine has the best penetration rank followed by stavudine, didanosine and lamivudine [**17**]. Considering the same parameters (molecular weight and protein binding), nevirapine from the NNRTI class and indinavir from the PI class have the best probability of reaching good CSF levels.

**Zidovudine (ZDV)** has the best partition coefficient in the brain and cerebral tissue, reflecting the lipid solubility of the compound [**17**]. ZDV is substrate for PGP, MRP-4 and MRP-5 [**18**]. One of the first studies on ZDV in CSF, demonstrated that penetration of ZDV into the CSF appeared to be dose independent, which may be an explanation for the efficacy of low doses of ZDV in the prevention and treatment of HIV–related neurological diseases [**19**]. Since the beginning of the HIV epidemic, treatment with ZDV was associated with decreased HIV RNA loads in CSF, less alteration of the brain tissue and improvement of neurocognitive performance in children with HIV encephalopathy [**20**].

**Stavudine (d4T)** CSF stavudine concentrations reached or exceeded the mean concentration, producing 50% of the maximal effect in vivo (EC 50) for HIV. Oatp–2 like transporter has been implicated in its uptake [**21**]. ENT1 and ENT2 have been suggested as transporters for d4T on animal models [**22**].

**Abacavir (ABC)** has moderate plasma protein binding and lipid solubility which account for a good CSF penetration rank. Animal models have shown that ABC reaches the brain, but not the CSF by a non–saturable mechanism, meaning that its transport across the BBB is not influenced by other drugs [**23**]. In an pharmacokinetics study on paired plasma–CSF from 54 patients, Caparelli et. al demonstrated a mean CSF/plasma ratio for ABC of 36% that increased progressively with the augmentation of the doses, and showed that ABC penetrated into the CSF where it reached adequate concentrations which were able to inhibit the replication of HIV in the CNS [**24**]. Although a better CSF penetration compared to other antiretrovirals has been demonstrated for ABC, its lower penetration rate as compared to the corresponding free fraction indicated the possible existence of other active efflux mechanisms. In 2008, indeed, Giri et. al., demonstrated that PGP is the dominant transporter limiting the CNS penetration of ABC [**25**].

In animal models, **Didanosine (ddI)** was able to cross the BBB using saturable and non–saturable mechanisms [**26**]. ddI transport from the blood into the choroid plexuses involves an OATP 2–like transporter. In patients with HIV infection, ddI reaches relatively good CSF concentrations [**27**].

**Lamivudine (3TC)** movement across the blood–CSF barrier was examined in an isolated choroid plexus model, which showed low CSF accumulation of this molecule [**28**]. However, 3TC uptake from blood into the choroid plexus was significant, and it was facilitated by a digoxin–sensitive transporter. In the same study, 3TC had no major interactions with ABC. Based on rat models it is speculated that OCTs and probably OACT are involved in the cellular uptake of ZDV and 3TC [**29**].

**Tenofovir (TDF)** reaches a CSF concentration of only 4% of the plasma concentration, suggesting a passive or limited active transport and probably active efflux mechanisms from the CSF. These concentrations did not exceed IC50 for the wild–type; therefore, TDF is not suited for controlling CSF HIV replication [**30**]. However, studies on animal models demonstrated that although the transport of TDF in the CSF is minimal, the TDF precursor, PMPA, can reach the brain and accumulate in the choroid plexuses. These observations are related to the hydrophilic nature of PMPA and indicate the possible existence of a transporter at the choroid plexus site [**31**].

Important differences have been observed among the NNRTI class representatives, concerning the penetration in CNS. It has been demonstrated that penetrability of **Efavirenz (EFV)** in CSF is limited. The efflux mechanism described was the induction of the expression and function of PGP [**32**]. EFV also inhibits MRP–1, MRP–2 and MRP–3 in a concentration–dependent manner [**33**]. In a small study on 9 patients, EFV levels were constantly less than 1% (0.61%, range 0.26%–0.99%) of plasma levels [**34**]. However, in the same study, the authors found a mean EFV concentration of 35.1 nM (range 6.6–58.9 nM), that was above the IC95 of HIV wild type. This indicated that even if EFV was present in the CSF at low levels, these levels were effective in suppressing CSF viral levels when used in combination therapy. In a more recent report, Best et.al [**35**] have demonstrated that, even if EFV concentrations in CSF were only 0.5% of plasma concentrations they exceeded IC50 for the wild–type virus and were enough to inhibit the replication of HIV in the CSF.

**Nevirapine (NVP)** crosses well the BBB [**36**], and maintains concentrations in CSF that remain stable in time [**37**]. NVP had the highest CSF/plasma penetrability rate when compared to other drugs [**38**]. NVP has the highest penetrability rank in Letendre’s classification [**9**], and therefore can be used in patients with neurocognitive impairment [**8,39**].

**Protease inhibitors (PI)** have good lipid solubility, and therefore they were expected to concentrate adequately in the brain, but, in fact, the CSF concentrations of these drugs are low [**40**]. The explanations for the limited penetrability of PIs in the CSF are: the presence of efflux mechanisms (all the PIs are substrates for PGP) and high plasma protein binding (with the exception of Indinavir). Boosting with low–dose ritonavir enhances PIs’ penetrability into the CSF [**41,8**].

**Indinavir (IDV)** has the best CSF concentrations among all PIs [**42–44**]. This advantage can be explained by low protein–binding. Median concentration of IDV in CSF was 210 nmol/l [**45**], which is the threshold for IC95 in vitro. IDV is essentially the only PI that reaches CSF concentrations above IC95 [**46**] [**42**]. From a clinical point of view, the presence of IDV in CSF was associated with significant improvement of neurocognitive performances [**47**].

Concerning the other PIs, it has been demonstrated that their concentrations in CSF are enough to control viral replication although some of them concentrate poorly in CSF compared to plasma due to limited binding to plasma proteins, high molecular weight and efflux mechanisms. A good example regarding this issue, is boosted **Lopinavir (LPV/r) – Kaletra**, one of the best characterized PIs in terms of CSF penetrability. Although LPV/r has a CSF/plasma penetration rate of 0.22, its CSF levels are above IC50, [**48,49**]. Moreover, patients with monotherapy with LPV/r, and those with cART containing LPV/r [**50**] have a good decrease of HIV RNA in CSF and plasma and a reduction of the immune activation [**50,51**]. Because it penetrates the central nervous system in therapeutic concentrations and appears to reduce HIV replication in the central nervous system, LPV/r may benefit subjects who receive a diagnosis of or are at risk for HIV–associated neurocognitive disorders [**51**]. Similarly, **Amprenavir (APV)** boosted with low–dose RTV reaches CSF concentrations above IC50. In a study presented by Letendre et. al. [**52**], boosted or unboosted FPV had concentrations higher than IC50 with a CSF/plasma ratio of 0.45 – 1.30.

Some PIs like **Nelfinavir (NFV)**, **Saquinavir (SQV)**, **Ataznavir (ATV)** and **Tipranavir (TPV)** do not reach therapeutical concentrations in CSF [**9**]. CSF levels for NFV [**53**] and for SQV and RTV were below the detection limit [**54**]. For ATV, the CSF concentrations were highly variable and 100–fold lower than plasma concentrations, even with ritonavir boosting. CSF concentrations of ATV do not consistently exceed the wild–type IC50 of atazanavir and may not protect against HIV replication in the CSF [**55**].

In a recent study, Yilmaz et. al. [**56**] found detectable CSF **Darunavir (DRV)** levels in all the assessed patients. Most of them exceeded or remained in the same range as levels needed to inhibit the replication of the wild type virus, making it probable that DRV, at least to some extent, contribute to the suppression of HIV replication in the central nervous system.

The penetrability of **Enfuvirtide (T20)** in the CSF is negligible and therefore in clinical settings, where direct CNS drug exposure is crucial, this drug is not likely to directly contribute to the local therapeutic effect [**57**]. Cases of virological failure secondary to selection of resistance mutations to T20 in CSF with consecutive loss of viral suppression in plasma have been described [**58**]. Still, T20 is recommended for patients with HIV related neurocognitive impairment for its effect on the plasmatic pool, preferably in association with other three antiretrovirals with good CSF penetrability [**57,59**]. The motivation of this approach is due to the fact that antiretroviral treatment reduces immune activation with an indirect effect on the CNS in addition to the control of HIV replication in the plasma compartment [**60**]. Concerning the new antiretroviral drugs, data available to date suggest a good penetrability of integrase inhibitor and CCR5 co–receptors in the CSF.

**Raltegravir (RGV)** demonstrated a good passage into CSF. In a study published in 2009 by Yilmaz and her collaborators, 50% of the CSF samples exceeded IC95. In addition to contributing to the control of systemic HIV–1 infection, raltegravir reaches local inhibitory concentrations in CSF in most, but not all, patients [**61**]. In a similar study, Letendre [**62**] detected RGV levels above IC50 in all CSF samples of 21 patients and the median concentration of RGV in the CSF was 14.5 ng/mL (IQR 9.3 – 26.1, range 6.0 – 94.2). The median plasma concentrations were 260.9 ng/mL (IQR 72.0 – 640.4, range 17.8 – 4870). The CSF/plasma ratio was 5.8% (IQR 2.1%– 17.8%, range 1.0%– 53.5%). Based on these results, RGV can be used as a component of regimens with good CSF penetrability.

Studies regarding HIV tropism and coreceptors usage in the brain cells are limited. As microglia and monocyte–derived macrophages are the support of productive HIV infection in the CNS, and most likely play a major role in the development of HIV dementia, several studies were focused on the coreceptors used by these cells and they found that CCR5 is the main coreceptor used by HIV–1 isolated from the brain [**63–65**]. Tropism discordances between viral populations present in the CSF and in plasma have been observed. R5–tropic virus was usually found in the CSF [**66,67**]. Patients with advanced HIV disease [**68**] and especially those with HIV associated dementia had evidence of macrophage tropism and CCR5 use [**69,70**], but strains that use both CCR5 and CXCR4 co–receptors for cell entry have been identified in the brains of some individuals [**71,72**]. However, it seems that brain–derived Env’s still had preferential CCR5 usage [**72**].

The clinical consequences of virus populations with different tropism in specific body and cellular compartments as compared to the viral population in plasma are poorly understood [**73–75**]. Based on the observations that CCR5 receptors are mostly used in the brain of HIV–infected individuals and on studies that demonstrate that Maraviroc can reach adequate concentrations in CSF (above IC50) [**76,77**], the use of Maraviroc might be beneficial in patients with HIV–associated CNS diseases, especially in those with neurocognitive impairment. Nevertheless, clinical trials are needed to demonstrate the effectiveness of Maraviroc in the brain and in order to answer the question if the brain can be considered a potential reservoir of CXCR4 after exposure to CCR5 inhibitors.

As antiretroviral drugs have limited penetration into the CSF, other alternative mechanisms have been studied in order to limit the viral replication and to provide neuroprotection. The alternatives designed “to cheat” the diffusion or efflux mechanisms are either ineffective [**78**] or too expensive (i.e. nanotechnology) [**79,80**]. On the other hand adjunctive therapies, like valoproic acid, lithium [**81**], minocycline [**82–84**] and other small molecules showed encouraging results in terms of improving neurocognition but are lacking longitudinal follow–up studies [**85**]. For example, minocycline, a second–generation tetracycline derivative, that has increased penetration in CNS, has been shown to decrease both the virus load in the cerebrospinal fluid and the brain, as well as the severity of CNS disease in a simian immunodeficiency virus macaque model of HIV–associated neurological disease [**82**]. These, together with its immunomodulatory properties that reduce chronic activation and inflammatory processes may make it a promising adjuvant in active antiretroviral therapy.

### Summary and further research questions

The efficacy of antiretroviral regimens in the CNS should concern every physician involved in the care of HIV–infected patients. This has become a requirement, as HIV–associated neurocognitive impairment nowadays involves mostly mild and moderate forms. Therefore, the diagnosis of neurocognitive impairment in an early stage is needed in order to give an ART regimen with good CSF penetrability rank, which will presumptively assure a degree of neuroprotection.

Compartmentalization of HIV infection with genetic differences between plasma and CSF strains requires the quantification of HIV RNA and HIV resistance profile in both plasma and CSF and adjustment of ART regimen according to both results.

The evaluation of efficacy of an antiretroviral regimen in CNS sanctuary requires beyond awareness on the penetrability and resistance mutations of ART in CSF, considering other factors as modification of BBB (e.g. inflammation), drug interactions and co–morbidities. CNS is a compartment where HIV enters in the early stages of infection and can undergo neuro–adaptation. The unique structure of BBB and the existence of well–organized efflux mechanisms block or severely limit the access of ART. Approaching this sanctuary is possible by medication with good lipid solubility, low molecular weight, and low plasma protein–binding that cannot be accessed by efflux transporters (either by avoiding or by blocking them). However, further studies are needed to find an ideal drug to fulfil these criteria and assure a good balance between therapeutic effect and neurotoxicity.

Longitudinal studies are needed to clarify if a neuro–cART can be protective for neurocognitive impairment in adults but also in the developing brain of children.
